# 5,8-Dihy­droxy-2,2-dimethyl-12-(3-methyl­but-2-en­yl)pyrano[3,2-*b*]xanthen-6-one (brasixanthone B)

**DOI:** 10.1107/S2414314622011981

**Published:** 2023-01-06

**Authors:** Nurr Maria Ulfa Binti Seruji, Mohd Mustaqim Rosli, Vivien Yi Mian Jong, Thiruventhan Karunakaran, Mas Atikah Lizazman, Anita Marlina, Yanti Yana Binti Halid

**Affiliations:** a Centre of Applied Science Studies, Universiti Teknologi MARA Sarawak, 94300 Kota Samarahan, Sarawak, Malaysia; b X-ray Crystallography Unit, School of Physics, University Sains Malaysia, 11800 USM, Penang, ., Malaysia; c Centre of Drug Research, Universiti Sains Malaysia, 11800 USM, Pulau Pinang, Malaysia; d School of Chemical Sciences, Universiti Sains Malaysia, 11800 USM, Penang, Malaysia; e Research Centre for Chemistry, National Research and Innovation Agency, Indonesia; f Department of Chemistry, Faculty of Science, University of Malaya, 50603, Malaysia; University of Kentucky, USA

**Keywords:** crystal structure, brasixanthone B, calophyllum gracilentum, xanthone, pyrano ring, 3-methyl­but-2-enyl side chain

## Abstract

The title compound (trivial name brasixanthone B), C_23_H_22_O_5_, isolated from *Calophyllum gracilentum*, is characterized by a xanthone skeleton of three fused six-membered rings plus an additional fused pyrano ring and one 3-methyl­but-2-enyl side chain. The core xanthone moiety is almost planar, with a maximum deviation 0.057 (4) Å from the mean plane. In the mol­ecule, an intra­molecular O—H⋯O hydrogen bond forms an *S*(6) ring motif. The crystal structure features inter­molecular O—H⋯O and C—H⋯O inter­actions.

## Abstract 2

The crystal structure of brasixanthone B, a naturally occurring xanthone isolated from the stem bark of *Calophyllum gracilentum*, is reported. The mol­ecule is characterized by a xanthone skeleton of three fused six-membered rings plus an additional fused pyrano ring and one 3-methyl­but-2-enyl side chain.

## Graphical abstract

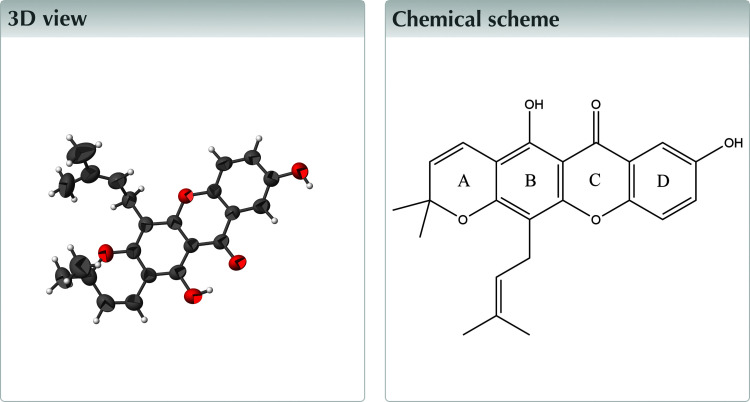

## Structure description

*Calophyllum*, frequently referred as ‘bintangor’ or ‘penaga’ in Malay is a part of the *Calophyllaceae* family (Crane *et al.*, 2005 ▸; Filho *et al.*, 2009 ▸). 80 different species have been identified in Malaysia (Corner, 1952 ▸), but studies on only 45 of them have been reported so far (Wang *et al.*, 2019 ▸). The ethnobotanical uses of *Calophyllum* in traditional medicine has been utilising several *Calophyllum* species for many thousands of years. This genus is well known for its medicinal uses and has been traditionally used for the treatment of potentially chronic diseases such as peptic ulcers, malaria, tumors, infections, high blood pressure, rheumatic disorders, eye infections, hemorrhoids, inflammation, malaria, and certain venereal diseases (Dweck & Meadows, 2002 ▸; Thia­garajan *et al.*, 2017 ▸; Zamakshshari *et al.*, 2019 ▸; Gupta & Gupta, 2020 ▸). For the biological activity of *Calophyllum* species, see: Guilet *et al.* (2001 ▸); Mah *et al.* (2012 ▸;) Reyes-Chilpa *et al.* (2004 ▸); Aminudin *et al.* (2015 ▸); Lim *et al.* (2017 ▸, 2019 ▸); Zamakshshari *et al.* (2019 ▸); Karunakaran *et al.*, 2022 ▸) and Gupta & Gupta (2020 ▸). Novel xanthones and coumarins are being identified from *Calophyllum* species on a regular basis (Aminudin *et al.*, 2015 ▸; Li *et al.*, 2016 ▸). The X-ray crystallographic structure for the title compound, brasixanthone B, isolated from *Calophyllum gracilentum* is reported herein. Related structures have been reported by Ito *et al.* (2002 ▸) and Mah *et al.* (2012 ▸).

The orientation of the 3-methyl­but-2-enyl side chain attached to ring *B* can be defined by the torsion angles C14—C15—C19—C20 and C16—C15—C19—C20, which have values of −85.2 (5) and 94.2 (4)°, respectively, suggesting a synclinal conformation (Ee *et al.*, 2010 ▸). Ring *A*, a 3,6-di­hydro-2*H*-pyran, forms a screw-boat conformation (Cremer & Pople, 1975 ▸), with puckering parameters *Q* = 0.352 (4) Å, θ = 65.4 (7)° and φ = 38.2 (7)°. The core xanthone moiety (rings *B*, *C* and *D*) is almost planar, with maximum deviation of 0.057 (4) Å from the mean plane for C16. The dihedral angles between xanthone rings are: 2.29 (19)° for *B* and *C*, 2.94 (19)° for *B* and *D*, and 0.75 (19)° between *C* and *D*. There are two methyl groups attached to atom C1 in ring *A* with C—C distances of 1.488 (6) and 1.483 (6) Å.

**Figure 1:**
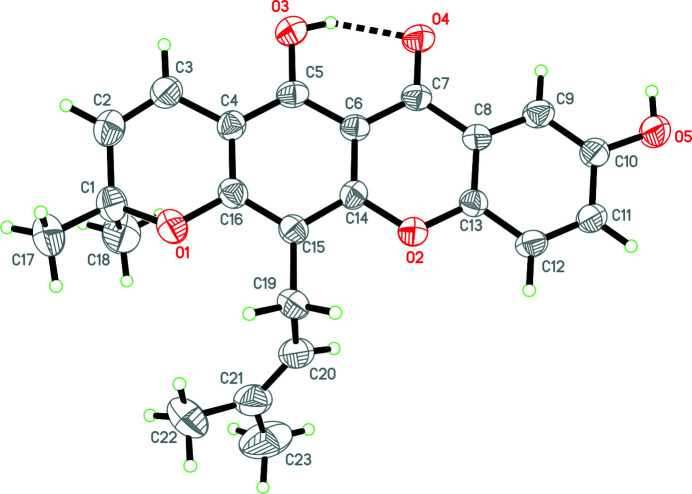
The mol­ecular structure of brasixanthone B showing the atomic labeling. Displacement ellipsoids are drawn at the 30% probability level. The intra­molecular hydrogen bond is shown as a dashed line.

**Figure 2:**
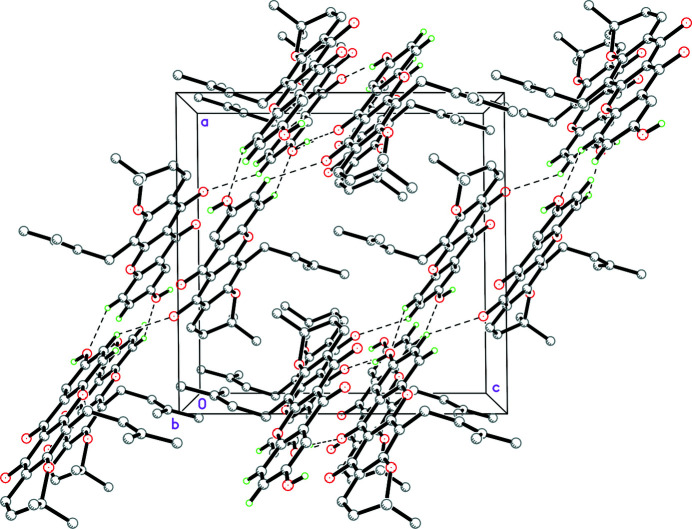
The crystal packing of brasixanthone B viewed along the *b*-axis direction. Hydrogen bonds are shown as dashed lines. Hydrogen atoms not involved in hydrogen bonds are omitted for clarity.

In the title compound (Fig. 1 ▸), an intra­molecular hydrogen bond, O3—H1*O*3⋯O4, forms an *S*(6) ring motif. In the crystal, the mol­ecules are linked by inter­molecular hydrogen bonds O5—H1*O*5⋯O4, C11—H11*A*⋯O3 and C12—H12*A*⋯O5 (Table 1 ▸), forming extended layers lying parallel to (101) (Fig. 2 ▸). Inversion-related (1 − *x*, 1 − *y*, 2 − *x*) mol­ecules are stacked by π–π inter­actions with an inter­planar spacing of 3.319 (4) Å between corresponding xanthone rings.

## Synthesis and crystallization

The stem bark (1.2 kg) of *calophyllum gracilentum* was ground and extracted with *n*-hexane, chloro­form, ethyl acetate and methanol. Fractionation of the hexane extract by gravity column chromatography over (Merck Kieselgel No. 1.09385.1000) silica gel with elution of *n*-hexa­ne: ethyl acetate and ethyl acetate: methanol in a step-wise gradual increment in polarity. This produced 28 fractions, which were combined and pooled together as 10 sub-fractions based on the TLC profile. Fraction 5 was subjected to further isolation by column chromatography over Sephadex LH20 eluted with methanol and several more purification steps using radial chromatography over silica (Merck Kieselgel No. 1.07749.1000), eluting with an *n*-hexa­ne:ethyl acetate (8:2) mixture. Yellow needle-like crystals were obtained. The melting point was found to be 500–502 K (Ee *et al.*, 2011 ▸).

## Refinement

Crystal data, data collection and structure refinement details are summarized in Table 2 ▸.

## Supplementary Material

Crystal structure: contains datablock(s) I. DOI: 10.1107/S2414314622011981/pk4037sup1.cif

Structure factors: contains datablock(s) I. DOI: 10.1107/S2414314622011981/pk4037Isup2.hkl

Supporting information file. DOI: 10.1107/S2414314622011981/pk4037Isup3.cml

CCDC reference: 2227328

Additional supporting information:  crystallographic information; 3D view; checkCIF report

## Figures and Tables

**Table 1:** Hydrogen-bond geometry (Å, °)

*D*—H⋯*A*	*D*—H	H⋯*A*	*D*⋯*A*	*D*—H⋯*A*
O3—H1*O*3⋯O4	0.82	1.77	2.520 (3)	152
O5—H1*O*5⋯O4^i^	0.82	1.89	2.679 (4)	160
C11—H11*A*⋯O3^ii^	0.93	2.58	3.352 (5)	141
C12—H12*A*⋯O5^iii^	0.93	2.50	3.352 (5)	152

Symmetry codes: (i) 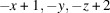 ; (ii) 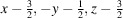 ; (iii) 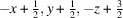 .

**Table 2:** Experimental details

Crystal data	
Chemical formula	C_23_H_22_O_5_
*M*_r_	378.40
Crystal system, space group	Monoclinic, *P*2_1_/*n*
Temperature (K)	296
*a*, *b*, *c* (Å)	13.071 (3), 10.458 (3), 13.358 (3)
β (°)	90.576 (19)
*V* (Å^3^)	1825.9 (8)
*Z*	4
Radiation type	Mo *K*α
μ (mm^−1^)	0.10
Crystal size (mm)	0.31 × 0.31 × 0.31

Data collection	
Diffractometer	Bruker APEX Duo CCD area detector
Absorption correction	Multi-scan (*SADABS*; Bruker, 2012 ▸)
*T*_min_, *T*_max_	0.773, 0.944
No. of measured, independent and observed [*I* > 2σ(*I*)] reflections	39924, 2381, 1409
*R*_int_	0.117
θ_max_ (°)	22.5
(sin θ/λ)_max_ (Å^−1^)	0.538

Refinement	
*R*[*F* ^2^ > 2σ(*F* ^2^)], *wR*(*F* ^2^), *S*	0.063, 0.198, 1.08
No. of reflections	2381
No. of parameters	257
H-atom treatment	H-atom parameters constrained
Δρ_max_, Δρ_min_ (e Å^−3^)	0.27, −0.24

Computer programs: *APEX2* and *SAINT* (Bruker, 2012 ▸), *SHELXT* (Sheldrick, 2015*a* ▸), *SHELXL2014/7* (Sheldrick, 2015*b* ▸), *SHELXTL* (Sheldrick, 2008 ▸) and *PLATON* (Spek, 2020 ▸).

## full crystallographic data

### Crystal data

**Table:**

C_23_H_22_O_5_	*F*(000) = 800
*M**_r_* = 378.40	*D*_x_ = 1.377 Mg m^−^^3^
Monoclinic, *P*2_1_/*n*	Mo *K*α radiation, λ = 0.71073 Å
*a* = 13.071 (3) Å	Cell parameters from 2238 reflections
*b* = 10.458 (3) Å	θ = 2.2–26.0°
*c* = 13.358 (3) Å	µ = 0.10 mm^−^^1^
β = 90.576 (19)°	*T* = 296 K
*V* = 1825.9 (8) Å^3^	Block, orange
*Z* = 4	0.31 × 0.31 × 0.31 mm

### Data collection

**Table:**

Bruker APEX Duo CCD area detector diffractometer	1409 reflections with *I* > 2σ(*I*)
Radiation source: fine-focus sealed tube	*R*_int_ = 0.117
φ and ω scans	θ_max_ = 22.5°, θ_min_ = 2.2°
Absorption correction: multi-scan (SADABS; Bruker, 2012)	*h* = −14→14
*T*_min_ = 0.773, *T*_max_ = 0.944	*k* = −11→11
39924 measured reflections	*l* = −14→14
2381 independent reflections	

### Refinement

**Table:**

Refinement on *F*^2^	0 restraints
Least-squares matrix: full	Hydrogen site location: mixed
*R*[*F*^2^ > 2σ(*F*^2^)] = 0.063	H-atom parameters constrained
*wR*(*F*^2^) = 0.198	*w* = 1/[σ^2^(*F*_o_^2^) + (0.1114*P*)^2^ + 0.0299*P*] where *P* = (*F*_o_^2^ + 2*F*_c_^2^)/3
*S* = 1.08	(Δ/σ)_max_ < 0.001
2381 reflections	Δρ_max_ = 0.27 e Å^−^^3^
257 parameters	Δρ_min_ = −0.24 e Å^−^^3^

### Special details

**Table:**

Geometry. All esds (except the esd in the dihedral angle between two l.s. planes) are estimated using the full covariance matrix. The cell esds are taken into account individually in the estimation of esds in distances, angles and torsion angles; correlations between esds in cell parameters are only used when they are defined by crystal symmetry. An approximate (isotropic) treatment of cell esds is used for estimating esds involving l.s. planes.
Refinement. The O-bound H atoms were located in a difference map, but fixed during refinement, with distance set to 0.82 Å and U_iso_(H) = 1.5U_eq_(O). The remaining H atoms were place in calculated positions with d(C-H) = 0.93 Å for aromatic, 0.97 Å for CH_2_ and 0.96 Å for CH_3_ atoms. The U_iso_(H) values were constrained to be 1.5U_eq_ of the carrier atom for methyl H atoms and 1.2U_eq_ for the remaining H atoms.

### Fractional atomic coordinates and isotropic or equivalent isotropic displacement parameters (Å^2^)

**Table:**

	*x*	*y*	*z*	*U*_iso_*/*U*_eq_	
O1	0.6393 (2)	0.7811 (2)	0.86169 (18)	0.0705 (8)	
O2	0.44525 (18)	0.4259 (2)	0.80181 (17)	0.0591 (7)	
O3	0.70808 (19)	0.4135 (2)	1.04401 (18)	0.0706 (8)	
H1O3	0.6883	0.3391	1.0424	0.106*	
O4	0.60038 (19)	0.2194 (2)	1.00573 (19)	0.0700 (8)	
O5	0.3386 (2)	−0.0672 (2)	0.8438 (2)	0.0850 (9)	
H1O5	0.3681	−0.1001	0.8918	0.127*	
C1	0.7421 (3)	0.8284 (4)	0.8784 (3)	0.0756 (12)	
C2	0.7815 (3)	0.7784 (4)	0.9731 (3)	0.0755 (12)	
H2A	0.8273	0.8272	1.0107	0.091*	
C3	0.7539 (3)	0.6671 (4)	1.0056 (3)	0.0694 (11)	
H3A	0.7841	0.6333	1.0632	0.083*	
C4	0.6778 (3)	0.5970 (4)	0.9535 (3)	0.0554 (10)	
C5	0.6545 (3)	0.4736 (4)	0.9740 (3)	0.0560 (10)	
C6	0.5765 (3)	0.4110 (3)	0.9236 (3)	0.0496 (9)	
C7	0.5540 (3)	0.2827 (4)	0.9415 (3)	0.0536 (10)	
C8	0.4738 (3)	0.2279 (4)	0.8831 (3)	0.0515 (9)	
C9	0.4461 (3)	0.1026 (4)	0.8922 (3)	0.0629 (11)	
H9A	0.4805	0.0505	0.9378	0.076*	
C10	0.3699 (3)	0.0538 (4)	0.8363 (3)	0.0624 (10)	
C11	0.3208 (3)	0.1285 (4)	0.7681 (3)	0.0634 (11)	
H11A	0.2690	0.0940	0.7282	0.076*	
C12	0.3464 (3)	0.2513 (4)	0.7580 (3)	0.0604 (10)	
H12A	0.3119	0.3026	0.7119	0.072*	
C13	0.4232 (3)	0.3012 (4)	0.8152 (3)	0.0517 (9)	
C14	0.5218 (3)	0.4796 (4)	0.8538 (3)	0.0515 (9)	
C15	0.5407 (3)	0.6040 (4)	0.8320 (3)	0.0533 (10)	
C16	0.6206 (3)	0.6586 (4)	0.8813 (3)	0.0573 (10)	
C17	0.7282 (4)	0.9696 (4)	0.8798 (4)	0.0996 (15)	
H17A	0.7940	1.0104	0.8830	0.149*	
H17B	0.6891	0.9933	0.9373	0.149*	
H17C	0.6928	0.9960	0.8200	0.149*	
C18	0.8048 (4)	0.7856 (5)	0.7928 (4)	0.1052 (16)	
H18A	0.8721	0.8217	0.7985	0.158*	
H18B	0.7734	0.8133	0.7313	0.158*	
H18C	0.8096	0.6940	0.7932	0.158*	
C19	0.4787 (3)	0.6755 (4)	0.7582 (3)	0.0663 (11)	
H19A	0.4081	0.6478	0.7629	0.080*	
H19B	0.4811	0.7657	0.7751	0.080*	
C20	0.5124 (3)	0.6594 (4)	0.6548 (3)	0.0754 (12)	
H20A	0.5130	0.5761	0.6304	0.091*	
C21	0.5412 (4)	0.7472 (6)	0.5937 (4)	0.0971 (16)	
C22	0.5441 (5)	0.8841 (6)	0.6180 (5)	0.150 (3)	
H22A	0.6108	0.9175	0.6039	0.225*	
H22B	0.5297	0.8957	0.6878	0.225*	
H22C	0.4938	0.9285	0.5784	0.225*	
C23	0.5722 (4)	0.7131 (7)	0.4911 (4)	0.150 (3)	
H23A	0.5340	0.7634	0.4437	0.225*	
H23B	0.5589	0.6240	0.4796	0.225*	
H23C	0.6439	0.7296	0.4834	0.225*	

### Atomic displacement parameters (Å^2^)

**Table:**

	*U*^11^	*U*^22^	*U*^33^	*U*^12^	*U*^13^	*U*^23^
O1	0.074 (2)	0.0613 (19)	0.0765 (19)	−0.0085 (14)	0.0017 (15)	0.0073 (14)
O2	0.0612 (16)	0.0555 (17)	0.0604 (16)	−0.0020 (13)	−0.0084 (13)	0.0054 (13)
O3	0.0690 (19)	0.0756 (19)	0.0669 (18)	−0.0041 (14)	−0.0158 (15)	0.0045 (14)
O4	0.0730 (19)	0.0682 (18)	0.0683 (18)	0.0026 (14)	−0.0207 (15)	0.0108 (14)
O5	0.099 (2)	0.0598 (19)	0.095 (2)	−0.0146 (15)	−0.0337 (17)	0.0113 (15)
C1	0.070 (3)	0.074 (3)	0.083 (3)	−0.017 (2)	0.005 (3)	0.006 (3)
C2	0.067 (3)	0.079 (3)	0.081 (3)	−0.011 (2)	0.000 (2)	−0.008 (3)
C3	0.068 (3)	0.075 (3)	0.065 (3)	−0.004 (2)	−0.004 (2)	−0.002 (2)
C4	0.049 (2)	0.062 (3)	0.055 (2)	−0.005 (2)	0.0009 (19)	−0.001 (2)
C5	0.050 (2)	0.067 (3)	0.052 (2)	0.009 (2)	−0.002 (2)	0.004 (2)
C6	0.046 (2)	0.054 (2)	0.049 (2)	0.0064 (18)	0.0004 (18)	0.0034 (19)
C7	0.051 (2)	0.059 (3)	0.051 (2)	0.0056 (19)	−0.001 (2)	0.006 (2)
C8	0.053 (2)	0.053 (2)	0.048 (2)	0.0031 (19)	−0.0023 (19)	0.0026 (19)
C9	0.067 (3)	0.059 (3)	0.063 (3)	0.007 (2)	−0.013 (2)	0.002 (2)
C10	0.068 (3)	0.051 (3)	0.067 (3)	0.001 (2)	−0.011 (2)	0.003 (2)
C11	0.066 (3)	0.062 (3)	0.062 (3)	−0.001 (2)	−0.013 (2)	0.001 (2)
C12	0.061 (3)	0.064 (3)	0.055 (2)	0.006 (2)	−0.011 (2)	0.004 (2)
C13	0.055 (2)	0.052 (3)	0.048 (2)	−0.0012 (19)	0.0014 (19)	0.0014 (19)
C14	0.047 (2)	0.055 (3)	0.053 (2)	−0.0028 (18)	−0.0011 (19)	−0.0026 (19)
C15	0.054 (2)	0.053 (2)	0.052 (2)	0.0002 (19)	0.0054 (19)	0.0014 (19)
C16	0.061 (3)	0.056 (3)	0.055 (2)	−0.003 (2)	0.006 (2)	0.003 (2)
C17	0.105 (4)	0.076 (3)	0.118 (4)	−0.027 (3)	−0.002 (3)	0.008 (3)
C18	0.104 (4)	0.123 (4)	0.089 (3)	−0.019 (3)	0.030 (3)	−0.004 (3)
C19	0.070 (3)	0.059 (3)	0.070 (3)	−0.002 (2)	−0.004 (2)	0.015 (2)
C20	0.075 (3)	0.085 (3)	0.066 (3)	0.002 (2)	−0.008 (2)	0.008 (3)
C21	0.086 (3)	0.128 (5)	0.078 (4)	−0.023 (3)	−0.012 (3)	0.034 (3)
C22	0.181 (6)	0.117 (5)	0.151 (6)	−0.050 (4)	−0.026 (5)	0.068 (4)
C23	0.122 (5)	0.263 (8)	0.065 (4)	−0.019 (5)	0.005 (3)	0.032 (4)

### Geometric parameters (Å, º)

**Table:**

O1—C16	1.330 (4)	C11—C12	1.335 (5)
O1—C1	1.446 (4)	C11—H11A	0.9300
O2—C14	1.336 (4)	C12—C13	1.360 (5)
O2—C13	1.348 (4)	C12—H12A	0.9300
O3—C5	1.321 (4)	C14—C15	1.357 (5)
O3—H1O3	0.8200	C15—C16	1.356 (5)
O4—C7	1.238 (4)	C15—C19	1.474 (5)
O5—C10	1.334 (4)	C17—H17A	0.9600
O5—H1O5	0.8199	C17—H17B	0.9600
C1—C2	1.458 (6)	C17—H17C	0.9600
C1—C18	1.483 (6)	C18—H18A	0.9600
C1—C17	1.488 (6)	C18—H18B	0.9600
C2—C3	1.295 (5)	C18—H18C	0.9600
C2—H2A	0.9300	C19—C20	1.464 (5)
C3—C4	1.414 (5)	C19—H19A	0.9700
C3—H3A	0.9300	C19—H19B	0.9700
C4—C5	1.354 (5)	C20—C21	1.287 (6)
C4—C16	1.374 (5)	C20—H20A	0.9300
C5—C6	1.382 (5)	C21—C22	1.468 (8)
C6—C14	1.371 (5)	C21—C23	1.477 (7)
C6—C7	1.395 (5)	C22—H22A	0.9600
C7—C8	1.421 (5)	C22—H22B	0.9600
C8—C13	1.355 (5)	C22—H22C	0.9600
C8—C9	1.365 (5)	C23—H23A	0.9600
C9—C10	1.340 (5)	C23—H23B	0.9600
C9—H9A	0.9300	C23—H23C	0.9600
C10—C11	1.357 (5)		
			
C16—O1—C1	118.1 (3)	O2—C14—C15	115.4 (3)
C14—O2—C13	119.8 (3)	O2—C14—C6	121.1 (3)
C5—O3—H1O3	105.6	C15—C14—C6	123.5 (4)
C10—O5—H1O5	108.3	C16—C15—C14	116.2 (3)
O1—C1—C2	109.3 (3)	C16—C15—C19	121.8 (4)
O1—C1—C18	107.3 (4)	C14—C15—C19	122.0 (4)
C2—C1—C18	111.5 (4)	O1—C16—C15	116.9 (3)
O1—C1—C17	103.2 (4)	O1—C16—C4	119.4 (4)
C2—C1—C17	112.7 (4)	C15—C16—C4	123.6 (4)
C18—C1—C17	112.2 (4)	C1—C17—H17A	109.5
C3—C2—C1	121.1 (4)	C1—C17—H17B	109.5
C3—C2—H2A	119.5	H17A—C17—H17B	109.5
C1—C2—H2A	119.5	C1—C17—H17C	109.5
C2—C3—C4	119.9 (4)	H17A—C17—H17C	109.5
C2—C3—H3A	120.1	H17B—C17—H17C	109.5
C4—C3—H3A	120.1	C1—C18—H18A	109.5
C5—C4—C16	117.9 (4)	C1—C18—H18B	109.5
C5—C4—C3	123.6 (4)	H18A—C18—H18B	109.5
C16—C4—C3	118.5 (4)	C1—C18—H18C	109.5
O3—C5—C4	118.6 (4)	H18A—C18—H18C	109.5
O3—C5—C6	120.2 (4)	H18B—C18—H18C	109.5
C4—C5—C6	121.3 (3)	C20—C19—C15	113.8 (3)
C14—C6—C5	117.4 (4)	C20—C19—H19A	108.8
C14—C6—C7	120.7 (4)	C15—C19—H19A	108.8
C5—C6—C7	121.9 (3)	C20—C19—H19B	108.8
O4—C7—C6	122.0 (4)	C15—C19—H19B	108.8
O4—C7—C8	121.2 (3)	H19A—C19—H19B	107.7
C6—C7—C8	116.7 (3)	C21—C20—C19	127.5 (5)
C13—C8—C9	118.3 (3)	C21—C20—H20A	116.3
C13—C8—C7	119.4 (4)	C19—C20—H20A	116.3
C9—C8—C7	122.2 (3)	C20—C21—C22	124.2 (5)
C10—C9—C8	120.8 (4)	C20—C21—C23	120.0 (6)
C10—C9—H9A	119.6	C22—C21—C23	115.7 (5)
C8—C9—H9A	119.6	C21—C22—H22A	109.5
O5—C10—C9	123.1 (4)	C21—C22—H22B	109.5
O5—C10—C11	117.0 (4)	H22A—C22—H22B	109.5
C9—C10—C11	119.9 (4)	C21—C22—H22C	109.5
C12—C11—C10	120.3 (4)	H22A—C22—H22C	109.5
C12—C11—H11A	119.8	H22B—C22—H22C	109.5
C10—C11—H11A	119.8	C21—C23—H23A	109.5
C11—C12—C13	119.8 (4)	C21—C23—H23B	109.5
C11—C12—H12A	120.1	H23A—C23—H23B	109.5
C13—C12—H12A	120.1	C21—C23—H23C	109.5
O2—C13—C8	122.2 (3)	H23A—C23—H23C	109.5
O2—C13—C12	117.0 (3)	H23B—C23—H23C	109.5
C8—C13—C12	120.8 (4)		
			
C16—O1—C1—C2	−43.1 (5)	C14—O2—C13—C12	−178.1 (3)
C16—O1—C1—C18	78.0 (4)	C9—C8—C13—O2	179.6 (3)
C16—O1—C1—C17	−163.3 (3)	C7—C8—C13—O2	−1.0 (5)
O1—C1—C2—C3	31.7 (6)	C9—C8—C13—C12	0.3 (5)
C18—C1—C2—C3	−86.9 (5)	C7—C8—C13—C12	179.7 (3)
C17—C1—C2—C3	145.9 (4)	C11—C12—C13—O2	−179.8 (3)
C1—C2—C3—C4	−5.5 (6)	C11—C12—C13—C8	−0.4 (5)
C2—C3—C4—C5	170.6 (4)	C13—O2—C14—C15	177.4 (3)
C2—C3—C4—C16	−12.8 (6)	C13—O2—C14—C6	−2.3 (5)
C16—C4—C5—O3	−179.3 (3)	C5—C6—C14—O2	180.0 (3)
C3—C4—C5—O3	−2.6 (5)	C7—C6—C14—O2	0.5 (5)
C16—C4—C5—C6	0.7 (5)	C5—C6—C14—C15	0.3 (5)
C3—C4—C5—C6	177.3 (3)	C7—C6—C14—C15	−179.1 (3)
O3—C5—C6—C14	178.2 (3)	O2—C14—C15—C16	−177.6 (3)
C4—C5—C6—C14	−1.7 (5)	C6—C14—C15—C16	2.1 (5)
O3—C5—C6—C7	−2.4 (5)	O2—C14—C15—C19	1.8 (5)
C4—C5—C6—C7	177.7 (3)	C6—C14—C15—C19	−178.5 (3)
C14—C6—C7—O4	−178.1 (3)	C1—O1—C16—C15	−155.5 (3)
C5—C6—C7—O4	2.4 (5)	C1—O1—C16—C4	28.4 (5)
C14—C6—C7—C8	1.0 (5)	C14—C15—C16—O1	−179.2 (3)
C5—C6—C7—C8	−178.4 (3)	C19—C15—C16—O1	1.4 (5)
O4—C7—C8—C13	178.4 (3)	C14—C15—C16—C4	−3.3 (5)
C6—C7—C8—C13	−0.8 (5)	C19—C15—C16—C4	177.3 (3)
O4—C7—C8—C9	−2.2 (5)	C5—C4—C16—O1	177.8 (3)
C6—C7—C8—C9	178.6 (3)	C3—C4—C16—O1	1.0 (5)
C13—C8—C9—C10	−0.8 (5)	C5—C4—C16—C15	2.0 (6)
C7—C8—C9—C10	179.8 (3)	C3—C4—C16—C15	−174.8 (3)
C8—C9—C10—O5	−178.6 (3)	C16—C15—C19—C20	94.2 (4)
C8—C9—C10—C11	1.4 (6)	C14—C15—C19—C20	−85.2 (5)
O5—C10—C11—C12	178.5 (4)	C15—C19—C20—C21	−121.4 (5)
C9—C10—C11—C12	−1.5 (6)	C19—C20—C21—C22	−0.8 (8)
C10—C11—C12—C13	1.0 (6)	C19—C20—C21—C23	−179.6 (4)
C14—O2—C13—C8	2.5 (5)		

### Hydrogen-bond geometry (Å, º)

**Table:**

*D*—H···*A*	*D*—H	H···*A*	*D*···*A*	*D*—H···*A*
O3—H1*O*3···O4	0.82	1.77	2.520 (3)	152
O5—H1*O*5···O4^i^	0.82	1.89	2.679 (4)	160
C11—H11*A*···O3^ii^	0.93	2.58	3.352 (5)	141
C12—H12*A*···O5^iii^	0.93	2.50	3.352 (5)	152

Symmetry codes: (i) −*x*+1, −*y*, −*z*+2; (ii) *x*−3/2, −*y*−1/2, *z*−3/2; (iii) −*x*+1/2, *y*+1/2, −*z*+3/2.
